# Prime factorization using quantum variational imaginary time evolution

**DOI:** 10.1038/s41598-021-00339-x

**Published:** 2021-10-21

**Authors:** Raja Selvarajan, Vivek Dixit, Xingshan Cui, Travis S. Humble, Sabre Kais

**Affiliations:** 1 Department of Chemistry, Department of Physics and Astronomy, and Purdue Quantum Science and Engineering Institute, Purdue University, West Lafayette, IN 47907 USA; 2 Department of Mathematics, Department of Physics and Astronomy, and Purdue Quantum Science and Engineering Institute, Purdue University, West Lafayette, IN 47907 USA; 3 Quantum Science Center Oak Ridge National Laboratory, Oak Ridge, TN USA

**Keywords:** Quantum mechanics, Quantum simulation

## Abstract

The road to computing on quantum devices has been accelerated by the promises that come from using Shor’s algorithm to reduce the complexity of prime factorization. However, this promise hast not yet been realized due to noisy qubits and lack of robust error correction schemes. Here we explore a promising, alternative method for prime factorization that uses well-established techniques from variational imaginary time evolution. We create a Hamiltonian whose ground state encodes the solution to the problem and use variational techniques to evolve a state iteratively towards these prime factors. We show that the number of circuits evaluated in each iteration scales as $O(n^{5}d)$, where *n* is the bit-length of the number to be factorized and *d* is the depth of the circuit. We use a single layer of entangling gates to factorize 36 numbers represented using 7, 8, and 9-qubit Hamiltonians. We also verify the method’s performance by implementing it on the IBMQ Lima hardware to factorize 55, 65, 77 and 91 which are greater than the largest number (21) to have been factorized on IBMQ hardware.

## Introduction

Quantum computation is likely to revolutionize how computation is performed in the field of science, engineering and finance. Computations are performed on quantum states that make use of superposition and entanglement to allow for speedups. Future potential applications include cryptography^1^, search problems^2^, simulation of quantum systems^3^, quantum annealing^4^, machine learning^5^, computation biology^6^, quantum materials^7^, and problems in optimization^8^. Refer^9^ for an extensive introduction into the field of quantum computation. Major efforts toward scaling the current algorithm focuses on developing error correction and mitigation schemes, as well as designing operations that make use of fewer ancilla qubits and gate operations^10^. In line with the current major developments, we investigate a more practical near-term scheme for prime factorization that is likely to achieve good results on noisy qubits.

Prime factorization involves expressing a composite number as the product of its prime factors. For generic large numbers that lack any structure, the quadratic sieve is the most commonly used technique. This can be computationally expensive and is exploited in RSA cryptography to guarantee information security over networks. From Shor’s^11^ work on period finding, it was shown that one could exploit the quantum Fourier transform to compute factors in steps that scaled polynomial in the number of bits. Subsequently, Vandersypen et al.^12^ realized this experimentally by factorizing $N=15$ using spin-1/2 nuclei as qubits and then Lucero et al.^13^ by using superconducting qubits. A simplified version of Shor’s algorithm was worked out by Geller et al.^14^for products of Fermat primes (3, 5, 17, 257 and 65537) . Later, Jian et al.^15^ proposed an alternative method to compute factors by solving an optimization problem using quantum annealing demonstrated on the D-Wave quantum annealer. The largest experimental realization of general method factorization schemes includes Shor’s algorithm to factorize 21^13^ and optimization using the D-Wave quantum annealer to factorize 223357^15^. In addition, large numbers with specific properties have also been factorized by exploiting structure contained in the number. In Ref.^16^, the authors use a multiplication table to factorize 56153 using only 4 qubits. Despite being able to factorize large numbers with relatively few qubits by exploiting the structure in the number, these methods do not reveal the power of quantum computers against generic numbers. Studies have not been made with respect to convergence on the solution with increasing the number of qubits.

In this paper we explore how one could use imaginary time evolution to factorize numbers with relatively higher probability. Imaginary time evolution has long been used as a theoretical tool in physics to compute ground-state wave-functions^17^. Shingu et al.^18^ show how using imaginary time evolution one could train a Boltzmann network efficiently, computing the exact model independent term in the training. Recently, Motta et al.^19^ exploits the Quantum Imaginary Time Evolution (QITE) algorithm to determine eigenstates and thermal states on a quantum computer. McArdle et al.^20^ showed how one could make use of variational circuits to create states that represent the dynamics of the imaginary time evolution. They use it to compute the ground state wavefunction of hydrogen and lithium hydride, while Yeter-Aydeniz et al.^21^ demonstrate that the QITE algorithm serves as a quantum computing benchmark for computational chemistry methods.

Herein, we develop an optimization function using the method introduced by Burges^22^ and then use it as a Hamiltonian to perform imaginary time evolution on a uniform superposition of all possible considered solutions to the factorization problem. We employ variational circuits to prepare a quantum state that encodes the solution and classically train all parameters. Simulations using Python Numpy packages and IBM-QASM are used to verify the performance. The robustness of the techniques against noise is verified on IBMQ hardware for up to 5 qubits allowing factorization of numbers up to 91 with a probability over $73\%$. To the best of our knowledge this is the largest number that has been factorized on a quantum circuit using a general purpose algorithm that does not exploit any structural properties of the number. Each iteration involves evaluating a number of circuits that scales as $O(n^5d)$ where *n* is the bit-string length of the number to be factorized and *d* is the circuit depth. A few reasons make factorization an ideal candidate to be solved using Imaginary time evolution with variational circuits. The number of terms in the Hamiltonian expansion scales polynomial with respect to the number of qubits. The amplitude of coefficients in the state vector is real, which simplifies the parameterization of the landscape to be explored and updates to be made. Each iteration only needs to amplify the amplitude of the solution rather than exactly simulate the imaginary time evolution. These factors make Variational QITE a good algorithm to be studied in the context of optimization, and make prime factorization a good test bed to evaluate its working. We are hoping that future rigorous work along these lines, with the availability of more less noisy qubits, might provide for ways in which this method complements with VQE for general optimization problems approached using variational methods.

## Background

The Schrödinger equation for a closed system describes dynamics according to some Hamiltonian that governs it. Allowing for time to be complex, we are able to create thermal states of specific temperature starting from a maximally mixed configuration, i.e, $\rho _{T=1/\tau } = e^{-H\tau }/$Tr$[e^{-H\tau }]$. Preparing a system at low temperature or alternatively letting the system evolve to large imaginary time values we can more often sample the ground state configuration for a given Hamiltonian. We shall make use of this property to encode the required solution of the problem we intend to solve in the later sections.

The quantum state we intend to prepare using QITE is given by,

1 $$\begin{aligned} \left| \psi (\tau )\right\rangle = N(\tau ) e^{-H\tau }\left| \psi (0)\right\rangle \end{aligned}$$

where $N(\tau )= 1/\sqrt{\left\langle \psi (0)\right| e^{-2H\tau }\left| \psi (0)\right\rangle }$ is a normalization factor. Equation (1) satisfies the Wick rotated Schrodinger equation

2 $$\begin{aligned} \frac{\partial \left| \psi (\tau )\right\rangle }{\partial \tau } = - (H -E_\tau ) \left| \psi (\tau )\right\rangle \end{aligned}$$

where $$E_{\tau }=\left\langle \psi (\tau )\right| H\left| \psi (\tau )\right\rangle$$.

Let $\left| \phi (\tau )\right\rangle$ be a state that is prepared by applying a series of unitary gates as follows

3 $$\begin{aligned} \left| \phi (\tau )\right\rangle = U_N(\theta _N)U_{N-1}(\theta _{N-1})U_{N-2}(\theta _{N-2})U_{N-3}(\theta _{N-3})\ldots U_1(\theta _1)\left| 0\right\rangle \end{aligned}$$

We choose the initial parameters to create the state on which the evolution is to be performed. We demand that Eq. (3) is an approximate solution to Eq. (2) by demanding the norm of the variation to vanish, i.e,

4 $$\begin{aligned} \delta \left| \left| \left( \frac{\partial }{\partial \tau } + H - E_\tau \right) \left| \phi (\tau )\right\rangle \right| \right| =0 \end{aligned}$$

Solving the above equation using McLachlan’s variational principle (see Supplementary section 1 for proof) we obtain

5 $$\begin{aligned} \sum A_{ij} \dot{\theta _j} = C_i \end{aligned}$$

where

6 $$\begin{aligned} \begin{aligned} A_{ij}= \frac{\partial \left\langle \phi (\tau )\right| }{\partial \theta _i}{\frac{\partial \left| \phi (\tau )\right\rangle }{\partial \theta _j}} \\ C_i = - \frac{\partial \left\langle \phi (\tau )\right| }{\partial \theta _i}H\left| \phi (\tau )\right\rangle \end{aligned} \end{aligned}$$

The derivatives on the state vectors with respect to the parameters of the circuit can be simplified into a sum of other basic circuit functions. For instance, the derivative of the $R_x$, $R_y$ and $R_z$ rotation gates with respect to their parameter amounts to a mere shift of the parameter by an angle of $\pi$ radians. We can further express *H* as a sum of a string of tensor products of Pauli operators allowing both *A* and *C* to be computed as the sum of terms that take the form $a {\mathfrak {R}}(e^{i\phi }\left\langle 0\right| U\left| 0\right\rangle )$, where *a* is some real number and $\phi$ is a real phase. Terms of this form can be efficiently computed on quantum hardware using the Hadamard test.

Evaluating the gradients using Eq. (5), we can then proceed to use gradient descent to update the parameters of the circuit as follows

7 $$\begin{aligned} {\vec {\theta }}(\tau +\delta \tau ) = {\vec {\theta }}(\tau ) +{ \dot{\vec {\theta }}}(\tau )\delta \tau \end{aligned}$$

where ${\dot{\vec {\theta }}}(\tau ) = A^{-1}(\tau )C(\tau )$. Using sufficient layers to the variational circuit and small $\delta \tau$ time iterations for updating our parameters, we can prepare states that closely emulate the imaginary time evolution on the state $\left| \psi (0)\right\rangle$. We would like to note that the dynamics of ITE is non unitary and thus we are only able to prepare the output state starting from a fixed initial state using variational methods.

## Methods

The problem of prime factorization involves expressing a number as a product of its primes. Here we shall focus specifically on biprimes, product of 2 prime numbers. Shor’s algorithm aims to solve this problem by reducing it to a period finding problem of the function $a^x$ mod *N*, where *a* is any random number and *N* is the number to be factorized. This algorithm uses a quantum subroutine that exploits the power of quantum Fourier transform as a part of a modified phase estimation algorithm. The largest number factorized using Shor’s algorithm is 21. Being highly sensitive to discrete errors, Shor’s algorithm fails to scale without viable error correction schemes^23^.

Here we show how to factorize a given number by first casting it as an optimization problem. Let the number *N* be given by a product of 2 prime numbers *p* and *q*. We model the problem of finding *p* and *q* given *N* as an optimization problem cast as the following cost function

8 $$\begin{aligned} H(p,q) = (N-p \times q)^2 \end{aligned}$$

Note that in Eq. (8), *H*(*p*, *q*) has global minima when *p*,*q* factorize *N* with a minimum value of 0. The cost function can thus be treated as a Hamiltonian whose ground state encodes the solution to our problem. We express both *p* and *q* as a binary string summation which will allow us to transfer to a qubit representation space. Assuming *p* has an *m* bit representation and *q* has an *l* bit representation, we get

9 $$\begin{aligned} \begin{aligned}p &= 2^{m-1}p_{m-1} + 2^{m-2}p_{m-2} \ldots 2^{0}q_{0} \\q &= 2^{l-1}q_{l-1} + 2^{l-2}q_{l-2} \ldots 2^{0}q_{0} \end{aligned} \end{aligned}$$

Note that Eq. (8) has a trivial solution $N=N \times 1$. To exclude this solution, we can restrict the search space of our solution by using a lower bound. The smallest prime factor we are looking for is greater than 2 and less than *N*/2. Thus using $N-1$ qubits to represent *p* and *q* we can discard the trivial solution from showing up in our simulation. Since both *p* and *q* are prime numbers, we are allowed to set the bit 0 to be 1 in either expression making it indivisible by 2. To move to a scaled spin representation we transform all the binary variables $b_i$ that represents either the $p_i$’s or $q_i$’s to $b_i = (s_i + 1)/2$. In this representation, $s_i$ takes the value $\pm 1$ such that $s_i=1$ maps to $b_i=1$ and $s_i=-1$ maps to $b_i=0$. We thus convert a factorization problem into an optimization problem over the spin variables.

Minimizing this optimization function shall result in the solution we seek. We use imaginary time evolution to evolve the output state starting from a uniform superposition over all possible solutions at $T=0$. The circuit parameters are updated according to Eqs. (5) and (7). With every iteration that is performed, the output state evolves through a time step of $\delta \tau$. After sufficiently large enough time *T*, we expect the ground state with the least cost to prepare. For $\left| \left| H\right| \right| _{1}T>> 1$ we get,

10 $$\begin{aligned} e^{-HT} \left| ++\cdots +\right\rangle \approx \left| \tilde{p}\right\rangle \left| \tilde{q}\right\rangle \end{aligned}$$

where $\tilde{p}$ and $\tilde{q}$ represent the bit string solution of the binary representation of the numbers that multiply to give *N*. The evolution is achieved by using a variational circuit ansatz to prepares states that mimic the evolution of the system from a given starting state (as described in the previous section). The computational cost in performing one iteration of QITE is $O(n^5d)$, where *n* is the bit-length of the number to be factored and *d* is the depth of the circuit (see Supplementary section 2 for proof).

Here we show an example of how to factorize $N = 15$ as

11 $$\begin{aligned} 15 = 5 \times 3 \end{aligned}$$

Expressing the factors 5 and 3 as binary variables and setting $p_0 =1$, $q_0 = 1$ yields

12 $$\begin{aligned} \begin{aligned}5 &= 4x_1 + 2x_0 + 1 \\3 &= 2x_2 + 1 \end{aligned} \end{aligned}$$

with the correct results defined by $x_0=0, x_1=1, x_2=1$. The corresponding cost function *H*(*p*, *q*) is cast as

13 $$\begin{aligned} H(x_0,x_1,x_2) = [15 - (4x_1 + 2x_0 + 1)(2x_2 + 1)]^2 \end{aligned}$$

Expanding the cost function and making use of $x_i^2 =x_i$ yields,

14 $$\begin{aligned} H(x_0,x_1,x_2) = 196 - 52 x_2 - 52 x_0 - 56 x_2 x_0 - 96 x_1 - 48 x_2 x_1 + 16 x_0 x_1 + 128 x_0 x_1 x_2 \end{aligned}$$

Mapping the binary variables to spin variables yields,

15 $$\begin{aligned} H(s_0,s_1,s_2) = 90 - 36 s_2 - 40 s_1 - 20 s_0 + 2 s_2 s_0 + 4 s_2 s_0+ 20 s_0 s_1 + 16 s_0 s_1 s_2 \end{aligned}$$

The classical binary variables $s_i$ in the Hamiltonian is replaced by the Pauli-z spin operator to get a quantum Hamiltonian Operator that is then acted upon the full superposition state. Using a time step $\delta \tau =0.1$ , we make 10 iterations of QITE to reach $T=1$. When simulated on QISKIT Aer using state-vector simulator backend, with a probability of greater than $90\%$, we obtain upon measurement

16 $$\begin{aligned} e^{-HT} \left| +++\right\rangle \approx \left| 110\right\rangle \end{aligned}$$

where the above expression is written in the computational basis. The output Eq. (16) when mapped back to the binary variables gives

17 $$\begin{aligned} x_0 = 0, x_1 = 1, x_2=1 \end{aligned}$$

We thus obtain the factors of 3 and 5 as expected. In our simulations, we shall make use of no more than the number of qubits needed to represent our solution in the optimization. This is to limit the numbers of qubits used, maximize the computational efficiency and reduce the accumulated errors.

**Figure 1:**
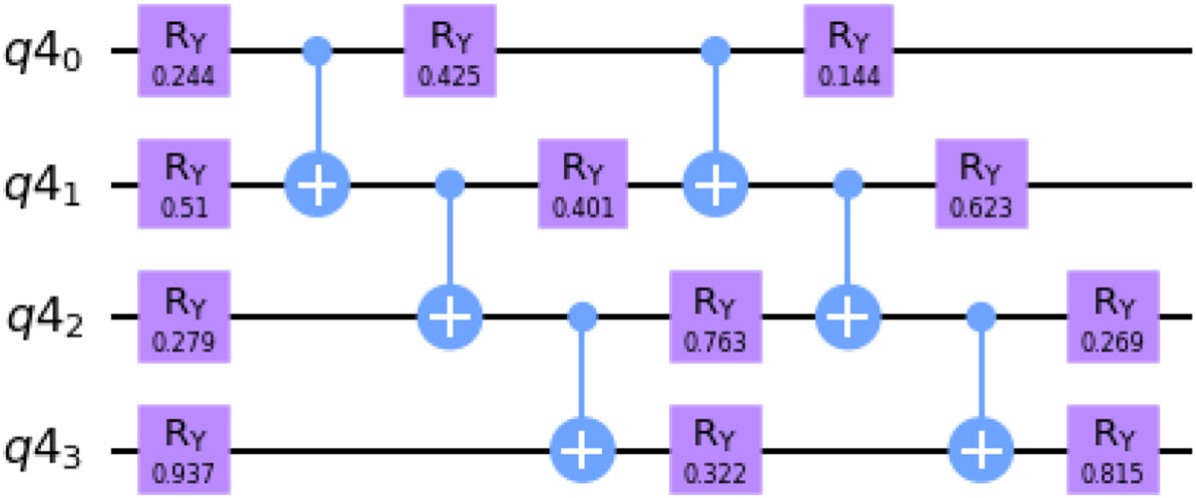
Variational circuit used to prepare the quantum state for imaginary time evolution. The parameters have been randomly initialized.

We generate the output quantum state prepared using QITE with the ansatz shown in Fig. 1. Notice that the circuit consists of $R_Y$ rotation gates applied to each qubit followed by a layer of *CNOT* gates that help with the entanglement of the qubits. Using only $R_Y$ gates ensures that we maintain real amplitudes for the state when expressed in the computation basis. This helps with closely following the dynamics of QITE without introducing any nontrivial phase values.

Equation (5) for such a circuit ansatz can be re-expressed in terms of the quantum Fisher information (QFI) and the gradient of Hamiltonian expectation with respect to the current state. In classical probability, the Fisher information characterizes how much a probability distribution varies by changing a parameter that characterizes a distribution. This can be generalized and extended to talk about quantum states where one characterizes how much a state changes with respect to the parameter that governs it. Refer^24^ for a brief introduction to quantum Fisher information. Given a quantum state $\left| \psi (\theta _1,\theta _2 \ldots \theta _n)\right\rangle$, where $\theta _i$ represents the governing parameters, the QFI is given by

18 $$\begin{aligned} F_{ij} = 4 {\mathfrak {R}}(\langle \psi _i|\psi _j\rangle -\langle \psi _i|\psi \rangle \langle \psi |\psi _j\rangle ) \end{aligned}$$

where $\left| \psi _j\right\rangle =\frac{d\left| \psi (\theta _j)\right\rangle }{d\theta }$ . For the ansatz being used to generate the state in QITE, we note that $\langle \psi _i|\psi \rangle = {\mathfrak {R}}(\langle \psi _i|\psi \rangle )$. This results in the second term vanishing due to the constant normalization of the state i.e, $\langle \psi |\psi \rangle =1$. Hence we get $F_{ij} = 4M_{ij}$ where $M_{ij} ={\mathfrak {R}}(\langle \psi _i(\theta (\tau ))|\psi _j(\theta (\tau ))\rangle )$. Note $F_{ij}$ being real symmetric, avoids the unstability that is likely to occur from inverting a skew symmetric matrix with small imaginary coefficients. Furthermore, since the Hamiltonian is an Hermitian operator, we can express the right hand side of Eq. (5) as follows

19 $$\begin{aligned} C_i = {\mathfrak {R}}(\left\langle \psi \right| H\left| \psi _i\right\rangle ) = \frac{d}{d\theta _i}(\left\langle \psi \right| H\left| \psi \right\rangle )/2 \end{aligned}$$

We thus obtain $\sum \nolimits _{j} F_{ij} \dot{\theta _j} = - 2\frac{d}{d{\theta }_{i}}\langle H\rangle _\psi$. The QFI of a given circuit and the gradient of an observable with respect to a state parameter can be directly computed in the IBM Qiskit Aqua framework (see Supplementary section 3 for QFI module).

## Results

### Simulations

**Figure 2:**
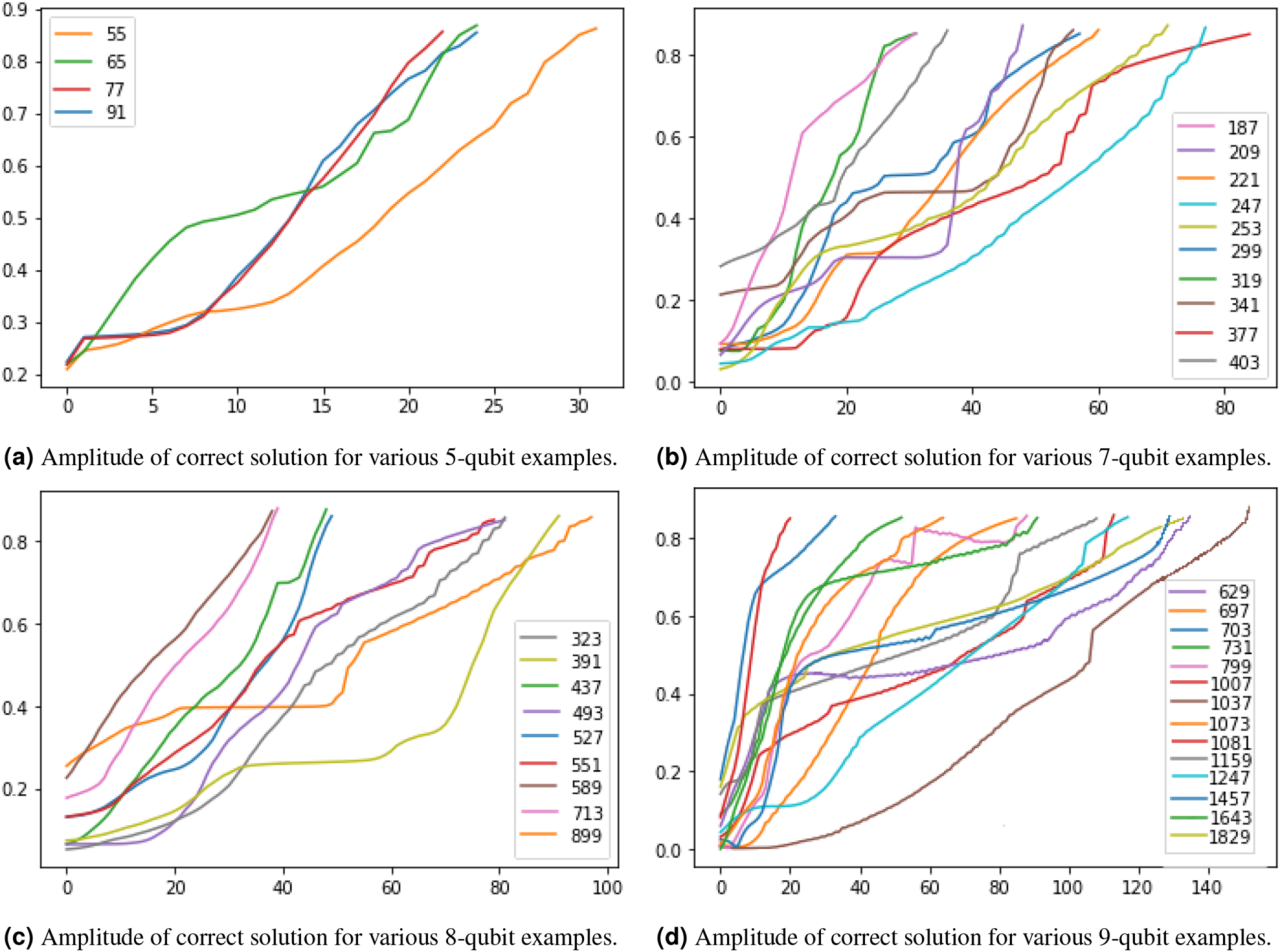
Numerical simulations of QITE for 7-, 8-, and 9-qubit factorization examples. The vertical axis indicates the amplitude of the solution in the computational basis and the horizontal axis indicates the number of iterations made. The curves in each subplot show how the amplitude of the solution corresponding to various numbers increases with iterations. The amplitude threshold has been set to 0.85.

Smaller numbers were factorized on Qiskit using the QASM simulator. For 8 qubits and more, the simulations were run using the Numpy package. The amplitude threshold for the correct solution has been set to 0.85, this amounts to a probability of $73\%$ to get the right solution when measured in the computational basis. We have restricted our ansatz to a single layer in addition to the base layer, in light of exploring shallow circuits for the near term quantum computers. The initial circuit parameters have been randomly sampled to help avoid valleys during the training and allow for faster convergence with shallow circuits employed. We have noticed that it also helps in suppressing one of the solutions in the case of symmetrically equivalent solutions (for instance, 391 can be factorized as $17 \times 23$ and $23 \times 17$), allowing one solution to quickly reach the threshold. Figure 2 plots the amplitude of the solution in the computational basis against the number of iterations for several values of *N* that are represented using 5, 7, 8, and 9-qubit Hamiltonians. Note the amplitude of the solution to the factorization keeps raising with increasing the number of iterations. The figure also shows that the average number of iterations required to factorize increases with the number of qubits though we have not been able to analytically bound it. A few instances involve the amplitude of the solution flattening before continuing to raise any further, making it significantly slow. In Table 1, we note that despite some numbers being close to each other (for instance 1007 and 1081), the number of iterations for convergence to the solution seem to be widely varying. This can be attributed to the differences in bit string representation and thus the point of minima to the created cost function, and also to the fact that the parameters are being randomly initialized. Results on actual hardware for smaller instances have been presented in the next subsection. We have tested this method against a standard Variational Quantum EigenSolver (VQE), and would like to note that no statistically significant difference with regards to the number of iterations for converging to the solution has been observed.

**Table 1:** Table lists the numbers that were factorized alongside the number of iterations for convergence.

Number factorized	Number of iterations
55	31
65	23
77	21
91	23
187	30
209	48
221	61
247	77
253	72
299	58
319	30
341	57
377	84
403	36
323	81
391	89
437	48
493	80
527	47
551	77
589	38
629	132
697	59
703	31
713	39
731	56
799	86
899	96
1007	21
1037	155
1081	112
1159	103
1247	112
1457	128
1643	88
1829	130

### Results on IBM-Hardware

**Figure 3:**
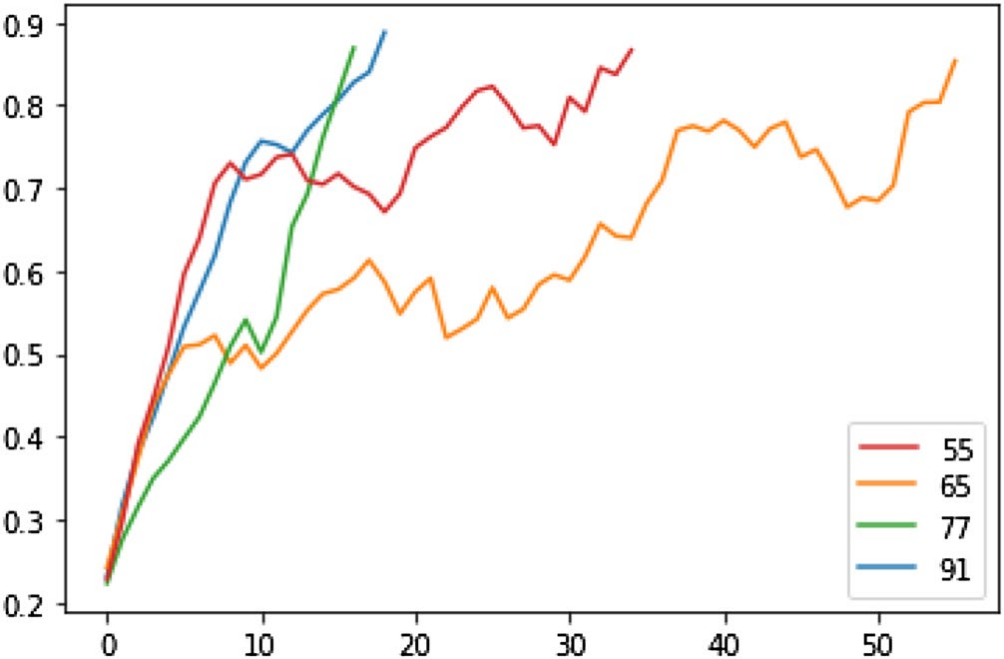
5-qubit factorization on IBMQ-lima. The vertical axis indicates the amplitude of the solution in the computational basis and the horizontal axis indicates the number of iterations made. The curves in each subplot, show how the amplitude of the solution corresponding to various numbers increase with iterations. The amplitude threshold has been set to 0.85.

We have made use of the publicly available IBM hardware to simulate factorization for up to 5 qubits. We start the output state with a uniform superposition over all possible solutions. This is straightforwardly instantiated by setting the parameters of $R_y$ gates in the base layer to be $\frac{\pi }{2}$ and the remaining parameters to be all 0. We have avoided invoking the Hadamard test to compute the *M* matrix and *C* vector as this resulted in significant error accumulation. Instead, we have reformulated the computation as indicated in the methods section and have made use of built-in Qiskit modules. The Gradient module with parameter shift method has been used to compute the *C* vector, while the QFI module with overlapping block diagonal method has been used to compute the *M* matrix. The built in QFI module makes 1024 shots on every circuit to be evaluated and includes no measurement error mitigation by default. Please refer to the Qiskit source documentation^25^ for details on the implementation of these modules. Figure 3 shows the results of factorizing the numbers $N =$ 55, 65, 77 and 91 on IBMQ-lima hardware that supports 5 qubits. Unlike the simulations that hardly show any oscillation in the amplitude, we see oscillations being introduced due to noise when run on the hardware. The convergence of the solution in the presence of hardware noise makes this method a suitable candidate for solving factorization and other similarly framed optimization problems in the near term quantum computers.

## Discussion

We have shown how imaginary time evolution can be used to perform optimization and have demonstrated this for the case of prime factorization. We have shown numerical and experimental results from the factorization of several numbers represented by 7, 8, and 9-qubit Hamiltonians. We have shown that imaginary time evolution significantly populates the probability of measuring the correct solution to the factorization problem when run sufficiently long. The observed performance of the method on the IBM-lima hardware shows that the method is robust to noise and works as a proof of concept in the case of a limited number of qubits. Future work towards bounding the number of iterations analytically can help highlight the performance of this method against well known factorization techniques. We hope that the results explored here promote future work in the direction of exploring Quantum Imaginary time evolution in the context of solving optimization problems.

## Supplementary Information

Supplementary Information.
