# Supplementary material for: Prime factorization using quantum variational imaginary time evolution
Source: Sci Rep. 2021 Oct 21;11:20835. doi: 10.1038/s41598-021-00339-x (PMC8531006; doi:10.1038/s41598-021-00339-x)
Supplement: Supplementary file 1 — Supplementary Information. [file 41598_2021_339_MOESM1_ESM.pdf]

Supplementary Material: Prime factorization using Quantum Variational Imaginary Time Evolution

1 McLachlan's Principle

We start from the variation of norm being set to zero and show how the parameters of the circuit that generate the state $|\psi(\tau)\rangle$ needs to be updated

$$\delta \left\| (\frac{d}{d\tau} + A) |\psi(\tau)\rangle \right\| = 0 \tag{1}$$

Here we take A to be Hermitian, which is true of the Hamiltonian. Working with the variation of the norm square,

$$\left\| (\frac{d}{d\tau} + A) |\psi(\theta(\tau))\rangle \right\|^2 = ((\frac{d}{d\tau} + A) |\psi(\theta(\tau))\rangle)^* ((\frac{d}{d\tau} + A) |\psi(\theta(\tau))\rangle) \tag{2}$$

Taking θ to be real,

$$\begin{aligned} &= \langle \psi_i(\theta(\tau)) | \psi_j(\theta(\tau)) \rangle \dot{\theta}_i \dot{\theta}_j + \langle \psi_i(\theta(\tau))| A |\psi(\theta(\tau))\rangle \dot{\theta}_i \\ &\quad + \langle \psi(\theta(\tau))| A |\psi_i(\theta(\tau)\rangle \dot{\theta}_i + \langle \psi(\theta(\tau))| A^2 |\psi(\theta(\tau))\rangle \end{aligned} \tag{3}$$

Here we have used the notation $|\psi_i(\theta)\rangle = \frac{d|\psi(\theta)\rangle}{d\theta_i}$. Computing the variation for a small time interval we get,

$$\begin{aligned} =&(\langle \psi_i(\theta(\tau)) | \psi_j(\theta(\tau)) \rangle \dot{\theta}_j + \langle \psi_j(\theta(\tau))| \frac{d |\psi(\theta(\tau)\rangle}{d\theta_i} \dot{\theta}_j \\ &+ \langle \psi(\theta(\tau))| A |\psi_i(\theta(\tau)\rangle + \langle \psi_i(\theta(\tau))| A |\psi(\theta(\tau))\rangle) \delta\dot{\theta}_i \end{aligned} \tag{4}$$

Setting the variation to vanish for independent θ_i amounts to the coefficients vanishing, i.e we get.

$$\Re(\langle \psi_i(\theta(\tau)) | \psi_j(\theta(\tau)) \rangle) \dot{\theta}_j = -\Re(\langle \psi(\theta(\tau))| A |\psi_i(\theta(\tau)\rangle) \tag{5}$$

Substituting $A = H - \langle \psi(\theta)| H |\psi(\theta)\rangle$ and the fixed normalization of the state giving that $\Re(\langle \psi(\theta)| |\psi_i(\theta)\rangle) = 0$, we get,

$$\Re(\langle \psi_i(\theta(\tau)) | \psi_j(\theta(\tau)) \rangle) \dot{\theta}_j = -\Re(\langle \psi(\theta))| H |\psi_i(\theta(\tau)\rangle) \tag{6}$$

We thus get $M_{ij}\dot{\theta} = C_i$, where $M_{ij} = \Re(\langle \psi_i(\theta(\tau)) | \psi_j(\theta(\tau)) \rangle)$ and
$C_i = \Re(\langle \psi(\theta))| H |\psi_i(\theta(\tau)\rangle)$

2 Computations per iteration on QITE

Let the number to factorized be represented in n bits. Each of its factors will be upper bounded by an n bit representation. Our Hamiltonian thus takes the following form,

$$H = [N - (2^{n-1}p_{n-1} + .. + 2^0 p_0)(2^{n-1}q_{n-1} + .. + 2^0 q_0)]^2 \tag{7}$$

Expanding this Hamiltonian we get, a constant term, N^2 terms of the type $p_i q_j$, $N^2(N-1)/2$ terms of the form $p_i q_j q_k$, $N^2(N-1)/2$ terms of the form $p_i p_j q_k$ and $N^2(N-1)^2/4$ terms of the form $p_i p_j q_k q_l$. The constructed Hamiltonian thus contains $[n(n+1)/2]^2 + 1 \approx O(n^4)$ terms in it. Note that the cost of computing the coefficients is also of the same order, given the number of terms is of $O(n^4)$, if one were to ignore any simplifications that are to be carried.

If we restrict to a d layer Ry circuit, we have nd circuit parameter updates to compute in every iteration. This involves computing an M matrix of size nd and a C vector of size nd. Computing each entry in a C vector involves working with each term of the Hamiltonian independently and summing them up to the end, thus we have $O(n^5 d)$ circuits to evaluate for C and $O(n^2 d^2)$ circuits to evaluate for M. Taking the circuit depth to grow slower than n^3, we get a total of $O(n^5 d)$ circuits to compute every iteration. To keep the overall run time of QITE polynomial its thus important that we are able to achieve convergence to the correct solution in polynomial number of steps.

3 Quantum Fisher Information from QISKIT

Given a quantum state $|\psi(\theta_1, \theta_2 ... \theta_n)\rangle$, where θ_i represents the governing parameters, the QFI is given by

$$F_{ij} = 4\Re(\langle\psi_i|\psi_j\rangle - \langle\psi_i|\psi\rangle\langle\psi|\psi_j\rangle) \tag{8}$$

Here we have used the notation $|\psi_i(\theta)\rangle = \frac{d|\psi(\theta)\rangle}{d\theta_i}$. The default QFI module built in QISKIT supports three methods: *lin_comb_full*, *overlap_block_diag*, *overlap_diag*. The 3 methods refer to the different approximations in rewriting the circuit as a sum of pauli strings to help with computing expectation values. The module comes with an added gradient wrapper that takes as input an *operator* (the circuit that builds the output state), *grad_params* (the parameters that are to be varied in the computation of the fisher information) and *backend* (the device on which the computation is to be executed). By default the number of shots made is 1024 and there isnt any inbuilt measurement error mitigation used by the module. Expectation values are computed by acting the pauli string operator on the state and doing statistics on the output measurements. The QFI module plays an important role within the natural gradient optimizer for minimizing distances between probability distributions as opposed to standard gradient descents that optimize on a standard euclidean norm.
